# Synthesis and activity of a diselenide bond mimetic of the antimicrobial protein caenopore-5†
†Electronic supplementary information (ESI) available. See DOI: 10.1039/c5sc04187b


**DOI:** 10.1039/c5sc04187b

**Published:** 2015-12-07

**Authors:** Karima Medini, Paul. W. R. Harris, Ayana Menorca, Kiel Hards, Gregory. M. Cook, Margaret. A. Brimble

**Affiliations:** a Maurice Wilkins Centre for Molecular Biodiscovery , School of Biological Sciences , The University of Auckland , 3A Symonds St , Auckland 1010 , New Zealand . Email: m.brimble@auckland.ac.nz ; Fax: +64 9 3737422 ; Tel: +64 9 3737599; b School of Chemical Sciences , The University of Auckland , 23 Symonds St. , Auckland 1010 , New Zealand; c Department of Microbiology and Immunology , School of Medical Sciences , University of Otago , 720 Cumberland Street , Dunedin 9054 , New Zealand

## Abstract

Antimicrobial proteins are a rich source of new lead compounds for the development of new drugs that will tackle global resistance towards existing antibiotics. Caenopore-5 (**Cp-5**) is an antimicrobial protein (AMP) expressed in the intestine of the nematode *Caenorhabditis elegans* and is a member of the lipid binding saposin-like-protein family, composed of 5 α-helices and 3 disulfide bonds. Substitution of the ^7^Cys and ^81^Cys by two selenocysteine ^7^U and ^81^U afforded a selenocysteine analogue **[^7^Sec-^81^Sec]-Cp-5**, which displayed a higher stability (using thermal circular dichroism) compared to the native protein **Cp-5**. **[^7^Sec-^81^Sec]-Cp-5** and an N-terminal truncated peptide exhibited cell permeability similar to the wild type **Cp-5**.

## Abstract 2

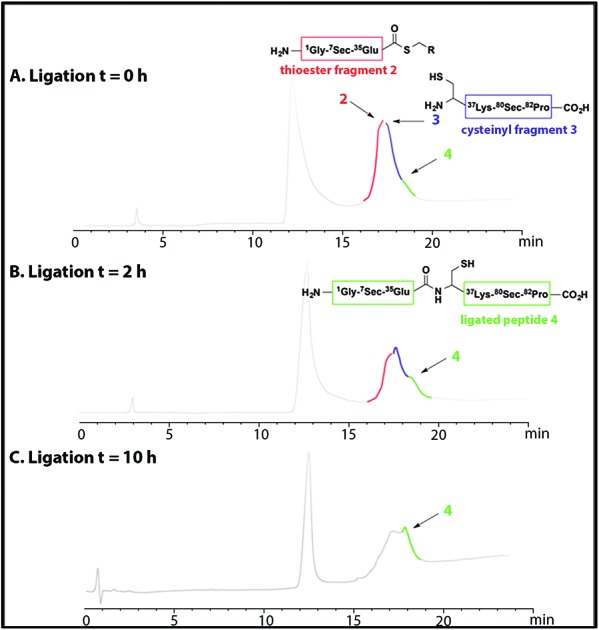
Antimicrobial proteins are a rich source of new lead compounds for the development of new drugs that will tackle global resistance towards existing antibiotics.

## Introduction

Antimicrobial resistance is a worldwide growing threat to the treatment of infections using antibiotics, due to multidrug-resistant (MDR) strains of pathogenic bacteria, parasites, viruses and fungi.^1^ Development of novel antimicrobial compounds to combat infections is therefore necessary. Over the last few decades, antimicrobial proteins (AMPs) have been investigated as alternatives to antibiotics.^2^,^3^ A wide variety of organisms: microorganisms, plants, invertebrates and vertebrates (including mammals), produce AMPs as part of their first line of defense.^4^–^6^ In addition, many AMPs are specific for their target cells, which is advantageous to avoid damage to the host cell or the commensal microbial flora.^4^–^7^

Caenopore-5 (**Cp-5**) is a pore forming AMP expressed in the intestine of the nematode *Caenorhabditis elegans*.^8^ The three-dimensional solution structure revealed that **Cp-5** is a member of the lipid binding saposin-like-protein (SAPLIP) family and is composed of 5 α-helices and 3 disulfide bonds.^9^ In our previous work, we reported a robust and efficient method for the chemical synthesis of native **Cp-5**, which would be amenable to the preparation of analogues.^10^ The 82-residue protein was successfully synthesized by native chemical ligation (NCL) of two smaller fragments (35 and 47 amino acids in length) and folded to give the correct protein structure. We also determined that the reduced form of **Cp-5** was inactive, thereby proving that the secondary structure is critical for the activity of the protein.^10^

Given the importance of developing peptides that display improved pharmacokinetic properties, efforts have been made to replace disulfide-bridges with more stable bonds such as thioether, carba and click variants.^11^ We herein report that replacement of a disulfide bond in **Cp-5** with a more robust diselenide bond resulted in more potent bioactivity and enhanced stability. Using our two fragment ligation strategy developed for the preparation of the native protein,^10^ the ^7^Cys and ^81^Cys were site selectively replaced with ^7^Sec and ^81^Sec respectively, and the linear protein was folded under redox conditions.

## Selenocysteine analogue of caenopore-5

Selenocysteine is used in a range of applications including the development of functional, structural and mechanistic probes, robust scaffolds, peptide conjugations, folding, and enzymatic reaction design.^12^,^13^ Selenocysteine can also be incorporated into peptide synthesis to prepare selenosulfide and diselenide-containing analogues.^13^–^15^ Selenocysteine has been shown to participate in native chemical ligation thus enabling access to selenium-containing proteins.^16^ To the best of our knowledge, the protein glutaredoxin 3 is the only example in the literature illustrating the synthesis of an intramolecular diselenide bond analogue of a native protein using standard NCL.^17^ With the goal of improving the stability and activity of **Cp-5**, ^7^Cys and ^81^Cys were substituted with selenocysteine, as these cysteine residues formed a disulfide bond, which is most exposed on the peptide surface and thus expected to be most susceptible to degradation.

### A. Synthesis of **[^7^Sec-^81^Sec]-Cp-5** (**1**)

**Scheme 1:**
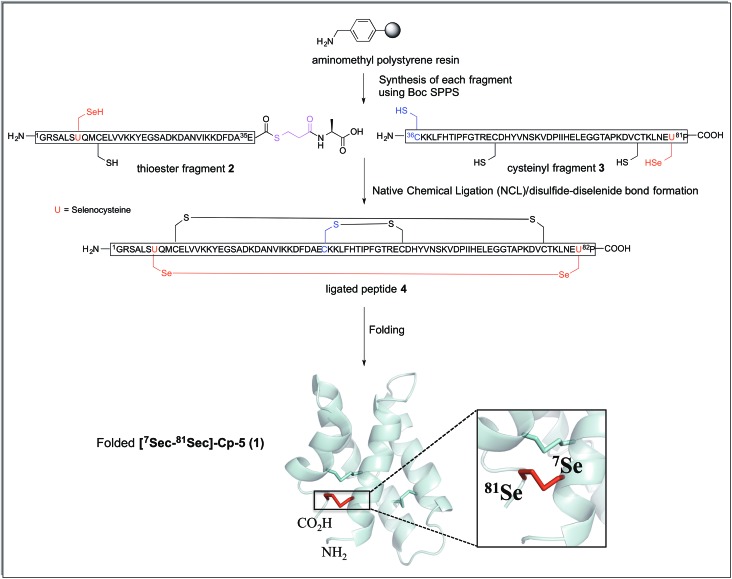
Synthetic strategy for the preparation of **[^7^Sec-^81^Sec]-Cp-5** (**1**). The connectivity of the ligated peptide **4** is the expected arrangement derived from the native protein **Cp-5**. The cartoon representation was generated from the solution structure of recombinant protein **Cp-5** by using the PDB ID: ; 2JS9.^9^

The synthetic strategy devised to prepare ^7^Sec-^81^Sec caenopore-5 analogue (**[^7^Sec-^81^Sec]-Cp-5**, **1**) was analogous to that described for our previous synthesis of the native protein.^10^ We adopted NCL using the native ^36^Cys residue as the ligation site, thereby avoiding introduction of a non-native cysteine that would require further chemical manipulation after the ligation step (Scheme 1). The requisite fragments, thioester fragment **2** (^1^Gly-^7^Sec-^35^Glu-COSCH_2_-CH_2_-Ala-OH)^18^ and cysteinyl fragment **3** (^36^Cys-^81^Sec-^82^Pro-COOH) were prepared using *in situ* Boc SPPS due to the sensitivity of thioesters to piperidine used in Fmoc SPPS, and to diketopiperazine formation when preparing C-terminal prolyl polypeptides. For the thioester fragment **2**, Boc-Ala-PAM was coupled to aminomethyl resin, followed by the attachment of *S*-trityl-3-mercaptopropionic acid giving TrtSCH_2_CH_2_-CO-Ala-PAM-resin. The resin bound *S*-trityl group was treated with TFA/TIPS/H_2_O (95 : 2.5 : 2.5 v/v/v) to unmask the thiol, which was then immediately reacted with the first amino acid of the sequence (^35^Glu) to form the thioester. For the cysteinyl fragment **3**, the synthesis was carried out using aminomethyl resin pre-loaded with Boc-Pro-PAM linker. For both fragments **2** and **3**, Boc amino acids were coupled using HATU/*i*Pr_2_EtN as activating agent and base, respectively.

Both fragments **2** and **3** were successfully synthesized in good yield (37% and 39%, respectively) after purification by HPLC. The purity (92% for thioester fragment **2** and 94% for cysteinyl fragment **3**) was confirmed by integration of the HPLC chromatogram at 210 nm and characterized by LC-MS [for **2**, (M + 5H)^+5^ calculated = 812.0 Da; (M + 5H)^+5^ observed = 811.6 Da and for **3** (M + 6H)^+6^ calculated = 889.6 Da; (M + 6H)^+6^ observed = 889.2 Da]. We note that the selenium-containing peptides were air sensitive and susceptible to oxidation. To overcome this problem, purified 1 mg aliquot of fragment **1** and **2** were prepared and lyophilized and stored at 20 °C which considerably reduced the oxidation of free selenol.

### B. Native chemical ligation

NCL of thioester fragment **2** with cysteinyl fragment **3** was carried out using the water soluble thiol M MPAA (100 mM) and 1–5 mM tris(2-carboxyethyl) phosphine (TCEP) or 1–5 mM dithiothreitol (DTT), under denaturing conditions at pH = 7. However, in the presence of the reducing agents TCEP and DTT, even at low concentration, degradation of both fragments was observed due to deselenization of the selenocysteine residues.^16^,^19^

**Fig. 1:**
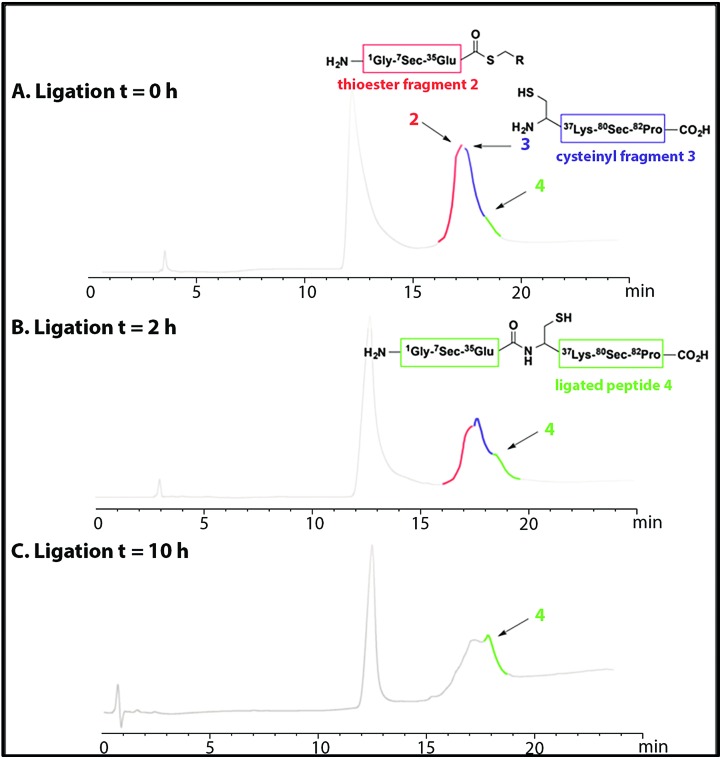
HPLC chromatograms showing the native chemical ligation of thioester fragment **2** (in red) and cysteinyl fragment **3** (in blue) at *T* = 0 h (A) and after 2 h (B). The ligated peptide **4** is shown in green. The ligation reached completion at *T* = 10 h (C).

Thus, to reduce the amount of thiol but to allow the ligation to occur the reaction was performed with thiophenol (2% (v/v)). Thiophenol is sparingly soluble under the aqueous ligation conditions however a hemiselenide bond with PhSH was observed as a significant by-product. This difficulty was outcome by reducing the amount of PhSH to 1% (v/v) with no detectable hemiselenide bond with PhSH was detected in this case. This method reduced the rate of the ligation reaction thus promoting the formation of undesired hydrolyzed thioester by-products, but this did not reduced the yield as more ligated product was observed. Hence, the successful conditions identified for the NCL of thioester fragment **2** with cysteinyl fragment **3** required use of 1% v/v PhSH (6 M Gd. HCl, 0.2 M Na_2_HPO_4_), with a 5 mM concentration of each fragment at pH 7.5 at 25 °C. The reaction was monitored at *T* = 0 h and *T* = 2 h, by LC-MS and formation of the ligated peptide **4** was observed (Fig. 1A and B) as suggested by mass spectrometry [(M + 10H)^+10^ observed = 921.2 Da; (M + 10H)^+10^calculated reduced = 922.0 Da], a 8 Da difference. This suggests that the protein may contain hemiselenides/diselenide/disulphide bonds that are formed by oxidation during the ligation reaction, which did not contain any reducing agent that could reverse unproductive oxidation. The ligation was deemed complete at *T* = 10 has no residual thioester fragment **2** was detected (Fig. 1C).

NCL conducted at elevated temperatures between 40–60 °C, was unsuccessful due to increased hydrolysis of the thioester fragment **2** (data not shown) and the yield of the NCL reaction was significantly reduced.

**Fig. 2:**
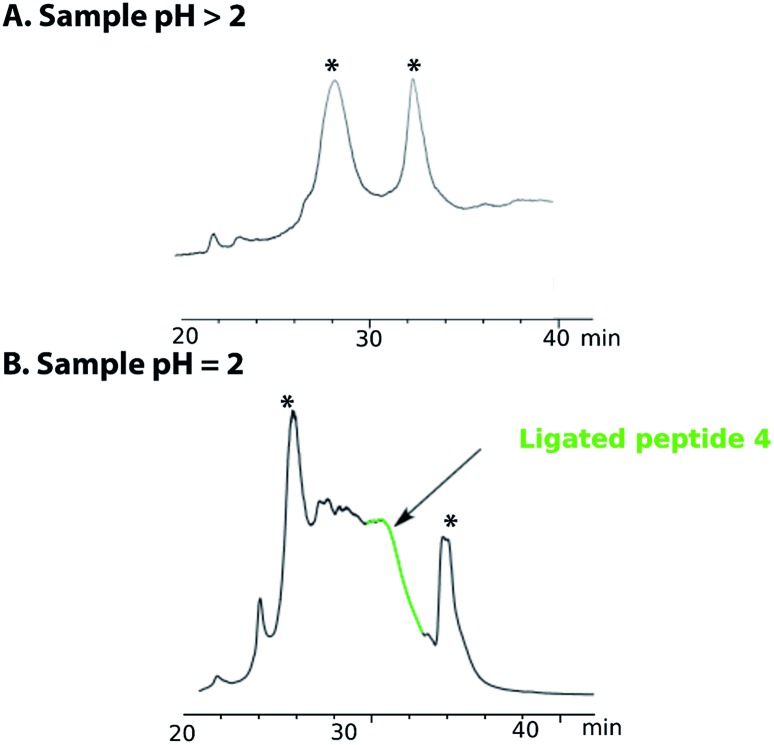
HPLC chromatogram of the purification of peptide **4** (A) when sample is quenched after ligation with a buffer solution at pH > 2 (5 or 3) and (B) at pH = 2. The peak annotated with * are non-peptide signal.

Purification of the ligated product **4** proved to be challenging as no reducing agent could be used to convert any premature oxidation back to the free thiols and/or selenium. On completion of the NCL reaction, the crude mixture containing the ligation product **4** was diluted with a 0.2 M sodium phosphate buffer and the pH was adjusted to either pH 5 or pH 3 with aqueous HCl. The sample was then loaded onto a C18 reverse phase column for purification (Fig. 2A). However, the ligated product was not detected in any of the fractions collected. Use of different stationary phases (C18, C3 and C8) at different temperatures (40–60 °C) afforded comparable results. However, when the crude ligation mixture containing the ligated peptide 4 was acidified to pH 2, the desired, polypeptide **4** (Fig. 2, highlighted in green) was separable from by-products and could be eluted from a C18 column (Fig. 2B). In spite of these difficulties, the purified peptide **4** was isolated in an acceptable yield of 32%.

**Fig. 3:**
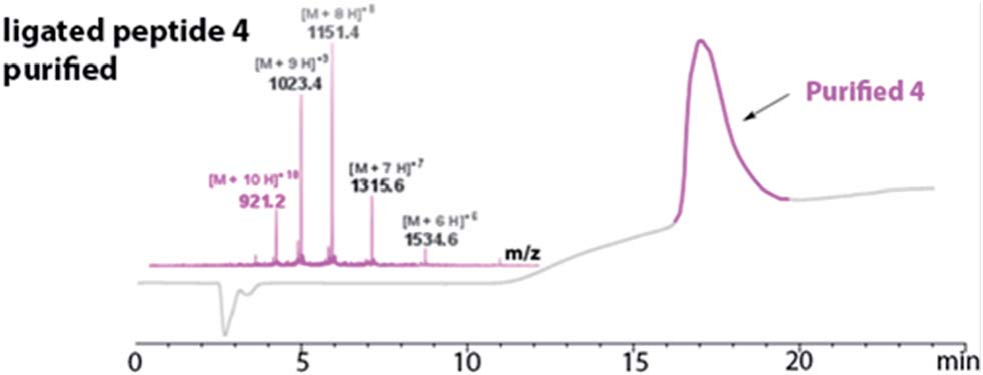
Purified peptide **4** using RP-HPLC (in pink). The LC-MS confirmed the purified peptide **4** [(M + 10H)^+10^ observed = 921.2 Da; (M + 10H)^+10^ calculated oxidized = 921.4 Da]. Deconvolution of the ESI-MS affords an observed mass of 9202.2 ± 0.62. The calculated mass is 9203.9.

The HPLC chromatogram of the purified peptide **4** is shown in Fig. 3. The broad elution profile suggested that **4** adopted several different conformations. Additionally, the mass of the purified ligated peptide **4** corresponded to the expected mass of the protein that contained one diselenide bond and two disulfide bonds. The deconvolution of the ESI-MS affords an observed mass of 9202.2 ± 0.62 Da for the purified ligated peptide **4** (Fig. 3). This compares favourably with the calculated mass of 9203.9 Da.

**Fig. 4:**
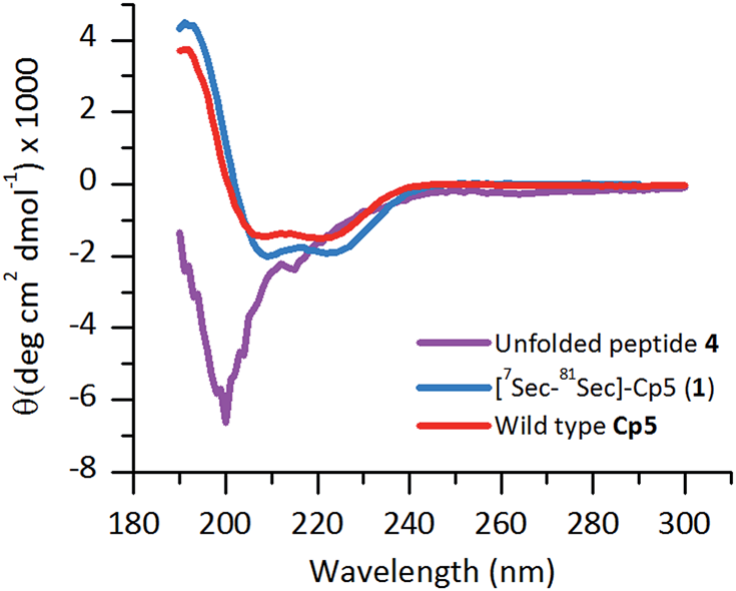
Far-UV CD-spectra of folded **[^7^Sec-^81^Sec]-Cp-5** (**1**) (blue) following purification by size exclusion chromatography. The unfolded ligated peptide **4** (purple), compared with the wild type protein **Cp-5** (red).

The ligated peptide **4** was characterized by circular dichroism (Fig. 4, in purple) and displayed a characteristic spectrum of an unstructured protein. Hence, the peptide **4** was subjected to folding.

### C. Folding and stability of **[^7^Sec-^81^Sec]-Cp-5**

To obtain the correctly folded **[^7^Sec-^81^Sec]-Cp-5** analogue (**1**), which contains two disulfide bonds and one diselenide bond, **4** was dissolved in the same folding buffer (50 mM Tris–HCl, 150 mM NaCl and 2 mM oxidized glutathione at pH 7.4, 4 °C) as empirically determined for recombinant **Cp-5** with the exception that reduced glutathione was omitted to avoid undesired reductive deselenization of the selenocysteine residues. After only 4 h, the folding was judged to be complete, and 5 was isolated in 17% yield. Introduction of ^7^Sec and ^81^Sec increased the rate of the folding step as it took 10–12 h to fold the native protein.^10^ This phenomenon was previously described by Alewood *et al.* Where they reported that the diselenide bond is formed first through selective oxidation and this subsequently directs assembly of the correct connectivity by reducing the number of intermediates involved in the folding process.^20^

Size-exclusion chromatography was used as the final purification step to isolate the folded **[^7^Sec-^81^Sec]-Cp-5** (**1**) as a monomer (see ESI Fig. S1†).

The purified folded **1** was studied by CD spectroscopy (Fig. 4, shown in blue) and compared with the recombinant protein **Cp-5** (Fig. 4, in red). They both exhibited the features expected of α-helical proteins, with standard double negative ellipticity maxima near 208 and 221 nm, and a positive maximum near 193 nm.

**Fig. 5:**
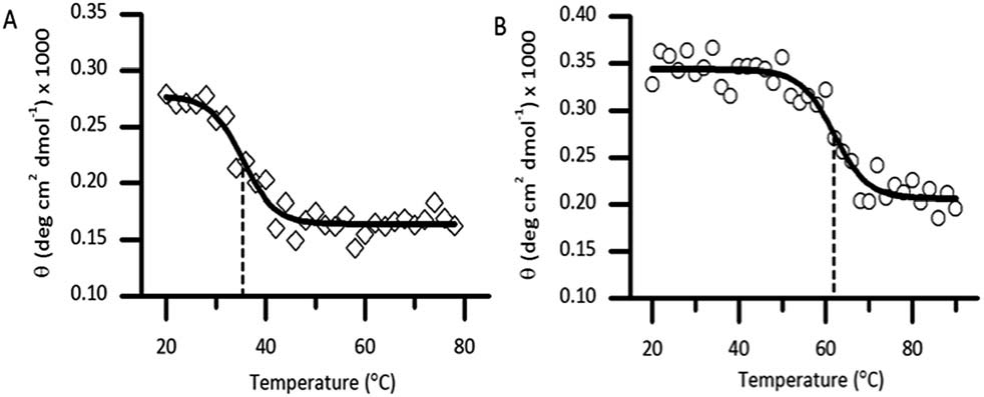
Denaturation of the native caenopore-5 ((A), white diamond) and the selenocysteine analogue **1** ((B), white circle) using thermal circular dichroism at 195 nm. The dashed line represents the *T*_M_ value.

Thermal unfolding using CD is widely used to determine the stability of proteins.^21^ Thermal CD obtained as a function of temperature was carried out to study the influence of the diselenide bond on the stability of the protein. The measurement of the midpoint of the unfolding transition (*T*_M_) of the folded analogue **1** provides insight into the stability when compared to the native protein **Cp-5** (Fig. 5A and B). The native **Cp-5** underwent denaturation at 35.39 ± 0.8 °C, while the selenocysteine analogue **1** underwent denaturation at a much higher temperature of 61.87 ± 0.97 °C. This data indicated a Δ*T*_M_ of 26.48 for analogue **1**, thus demonstrating that the presence of the diselenide bond significantly increases the stability of the protein against thermal denaturation. This is an unexpected result as the bond dissociation energy is weaker for diselenide bond (172 kJ mol^–1^) compared to a disulphide bond (226 kJ mol^–1^).^22^ This result suggests that the **[^7^Sec-^81^Sec]-Cp-5** analogue (**1**) might adopt a different bond connectivity and/or tertiary structure, which is more stable than the recombinant protein **Cp-5**.

### D. Permeability ability of **[^7^Sec-^81^Sec]-Cp-5** and analogues

**Fig. 6:**
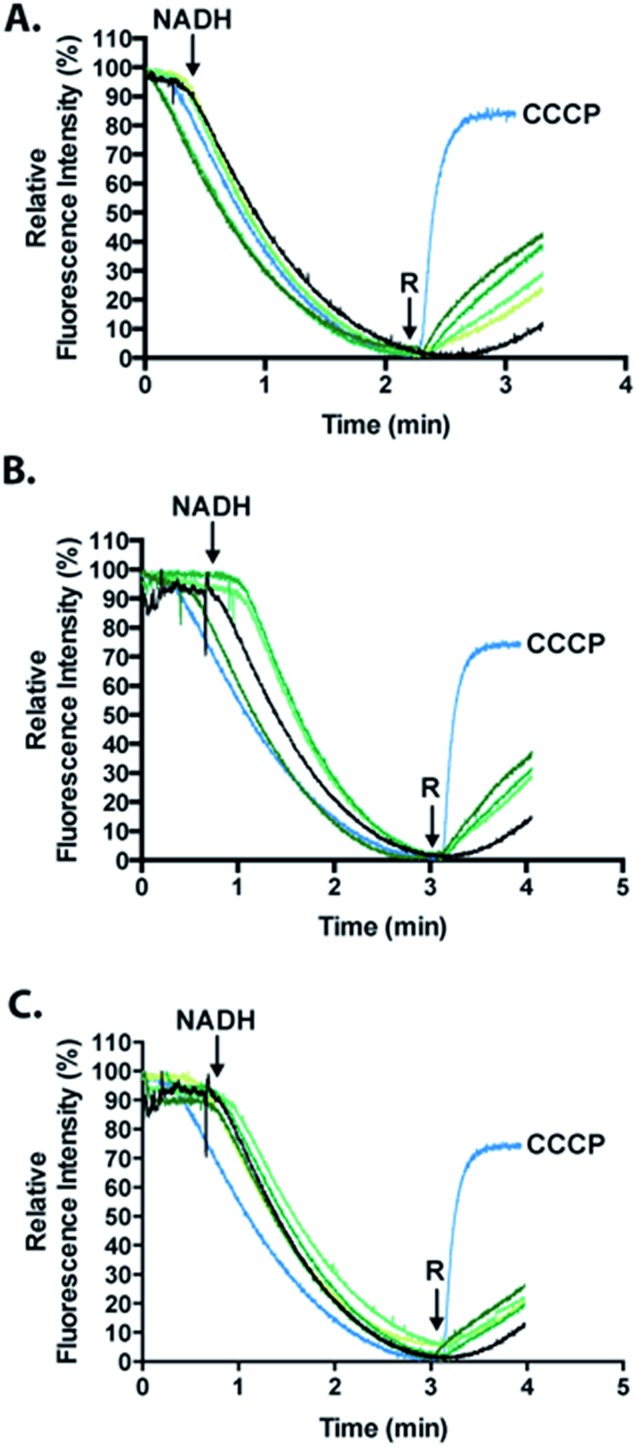
Measurement of the activity of the full length **[^7^Sec-^81^Sec]-Cp-5** (**1**, (A)); N-terminus (hydrolyzed **2A**, (B)) and C-terminus ((C), cysteinyl fragment **3**) using the fluorescence quenching experiments that is dependent on ΔpH. The relative fluorescence intensity (%) is shown as a function of time. The green gradients from light to dark correspond to 1, 2, 4 and 8 μM of peptide. The positive control, the carbonyl cyanide *m*-chlorophenyl hydrazone photo more (CCCP) is shown in light blue and the cells untreated are represented in black. *R* represents the time point when the compound was injected to induce a reverse quenching.

To analyze the permeabilization ability of **[^7^Sec-^81^Sec]-Cp-5** and analogues an assay that measures the difference in pH when *E. coli* MG1655 inverted membrane permeabilized was used. If a given compound can permeabilize a lipid bilayer, then it follows that chemical gradients previously established across that bilayer will be free to reach equilibrium through passive diffusion. The ability of the synthesized compounds to dissipate such gradients was assessed in order to infer its ability to permeabilize membranes. Acridine orange (AO) is a pH-responsive fluorophore and a lipophilic weak base routinely used for detecting proton gradients across lipid bilayers.^23^ Its unprotonated form can diffuse across membranes and distribute according to its p*K*_a_. The charged, protonated form cannot diffuse across membranes and does not fluoresce. Inverted membrane vesicles of *E. coli*, containing all of the organism's native membrane proteins and lipids, were allowed to generate a proton gradient (inside of vesicle lumen acidic) through proton pumping elicited by biological respiratory activity – as indicated by fluorescence quenching stimulated by NADH (Fig. 6). Compounds causing reversal of quenching from this state have dissipated the established proton gradient across the lipid membrane (*cf.* the protonophore CCCP positive control).

The inference made herein is that the synthesized compounds achieve this by destabilizing the membrane through its permeabilization activity.

Analogues of **Cp-5**, namely hydrolyzed fragment **2A**, (^1^Gly-^7^Sec-^35^Glu-OH, N-terminal region) and cysteinyl fragment **3** (^36^Cys-^81^Sec-^82^Pro-OH, C-terminal region) were also investigated as the N-terminal region of **Cp-5** is postulated to be responsible for membrane interaction.^10^ The peptides **[^7^Sec-^81^Sec]-Cp-5** (**1**) and hydrolyzed **2A** (^1^Gly-^7^Sec-^35^Glu-OH) were found to be effective at collapsing the pH (proton) gradient induced by NADH (Fig. 6, Table 1). The effect of the C-terminal peptide (cysteinyl fragment **3**) was less active in this assay (Table 1).

**Table 1:** Peptides tested with their % fluorescence after injection (to induce a reverse quenching) after subtraction of the baseline value (untreated) and relative to the quenching effect of the positive control, CCCP (*i.e.*, CCCP = 100%)

Peptide	% fluorescence at 8 μM
**[^7^Sec-^81^Sec ]-Cp-5** (**1**)	42.3
^1^Gly-^7^[Sec]-^35^Glu-OH (**2A**)	49.4
^36^Cys-^80^[Sec]-^81^Pro (**3**)	22.1

The relative fluorescence quenching reversal of the recombinant **Cp-5** was previously reported to be 35.2%, in an analogous assay to that performed here.^10^ In this work, the analogue **[^7^Sec-^81^Sec]-Cp-5** (**1**) was found to be able to reverse fluorescence quenching by 42.3% in the same time frame. This data suggests that substitution of ^7^Cys and ^81^Cys with ^7^Sec and ^81^Sec affords an analogue **[^7^Sec-^81^Sec]-Cp-5** (**1**) with improved activity compared to recombinant **Cp-5**. This increase in activity might be explained by the greater overall protein stability resulting from substitution of the disulfide bond for a more robust diselenide bond. To our knowledge, this is only the second example of using NCL to form an intramolecular diselenide bond affording a diseleno analogue of the native protein, which retained its secondary structure and activity.

## Conclusions

This paper reports the successful synthesis of a diselenide-bond analogue of the antimicrobial protein, caenopore-5, which contains one diselenide bond and two disulfide bonds. Substitution of the ^7^Cys-^81^Cys disulfide bond by a diselenide bond resulted in the disulphide and diselenide bonds being observed directly during the ligation reaction although the protein was unstructured. A separate folding step affording structured **[^7^Sec-^81^Sec]-Cp-5** (**1**) analogue, which maintain its permeability towards cell membranes. **[^7^Sec-^81^Sec]-Cp-5** (**1**) unexpectedly displayed increased stability as determined by thermal circular dichroism when compared to wild type **Cp-5**. To unravel the reasons for this increased stability, we are currently exploring X-ray crystallography methods to determine the 3-D structure of **[^7^Sec-^81^Sec]-Cp-5**.

## Supplementary Material

Supplementary information
