# Supplementary material for: Synthesis and activity of a diselenide bond mimetic of the antimicrobial protein caenopore-5
Source: Chem Sci. 2015 Dec 7;7(3):2005–10. doi: 10.1039/c5sc04187b (PMC5968448; doi:10.1039/c5sc04187b)
Supplement: Supplementary file 1 [file SC-007-C5SC04187B-s001.pdf]

Electronic Supplementary Material (ESI) for Chemical Science.
This journal is © The Royal Society of Chemistry 2015

Supporting Information

Synthesis and Activity of a Diselenide Bond Mimetic of the Antimicrobial Protein Caenopore-5.

Karima Medini, Paul. W. R. Harris, Ayana Menorca, Kiel Hards, Gregory. M. Cook, Margaret. A. Brimble.

1. General Experiential.

All solvents and reagents were used as supplied. *O*-(Benzotriazol-1-yl)-*N,N,N',N'*-tetramethyluronium hexafluorophosphate (HBTU), (*O*-(7-azabenzotriazol-1-yl)-*N,N,N',N'*-tetramethyluronium hexafluorophosphate) (HATU), benzotriazol-1-yloxy)tris(dimethylamino)phosphonium hexafluorophosphate (BOP), benzotriazol-1-yloxy)tripyrrolidinophosphonium hexafluorophosphate (PyBOP) and S-trityl mercaptopropionic acid were purchased from GL Biochem (Shanghai, China). Dimethylformamide (DMF) (AR grade) and acetonitrile (HPLC grade) were purchased from Scharlau (Barcelona, Spain). *N, N'* diisopropylethylamine (DIPEA), piperidine, ethanedithiol (EDT), diisopropylcarbodiimide (DIC), triisopropylsilane (TIS), were purchased from Aldrich (St Louis, MO) and *N*-methylpyrrolidine (NMP) was purchased from Fluka (Buchs, Switzerland). TFA was purchased from Halocarbon (River Edge, NJ). Anhydrous hydrogen fluoride was obtained from Matheson Trigas (Basking Ridge, NJ). Aminomethyl polystyrene (AM-PS) resin was synthesised "in house" as described. Boc-Ala-PAM (PAM = phenylacetamidomethyl) linker and Fmoc-Glu(tBu)-HMPP (HMPP = hydroxymethylphenoxypropionic acid) was purchased from Polypeptides (Strasbourg, France). Fmoc-amino acids were purchased from GL Biochem with the following side chain protection: Fmoc-Arg(Pbf)-OH (Pbf = 2,2,4.6,7-pentamethyldihydrobenzofuran-5-sulfonyl), Fmoc-Asn(Trt)-OH (Trt = triphenylmethyl), Fmoc-Asp(tBu)-OH (tBu = *tert*-butyl), Fmoc-Cys(Trt)-OH, Fmoc-Gln(Trt)-OH, Fmoc-Glu(tBu)-OH, Fmoc-His(Trt)-OH, Fmoc-Lys(Boc)-OH, Fmoc-Ser(tBu)-OH, Fmoc-Thr(tBu)-OH, Fmoc-Tyr(tBu)-OH and Fmoc-Trp(Boc)-OH. Boc-amino acids were purchased from Polypeptides with the following side chain protection: Boc-Arg(Tos)-OH (Tos = *p*-toluenesulfonyl), Boc-

Asp(cHex)-OH (cHex = cyclohexyl), Boc-Cys(4-MeBn)-OH (Bn = benzyl), Boc-Asn(Xan)-OH (Xan = Xanthyl), Boc-Glu(cHex)-OH, Boc-His(Bom)-OH (Bom = benzyloxymethyl, Boc-His(Tos)-OH.DCHA (DCHA = dicyclohexylamine), Boc-Lys(2-Cl-Z)-OH (Z = benzyloxycarbonyl), Boc-Ser(Bn)-OH, Boc-Thr(Bn)-OH, Boc-Tyr(2-Br-Z)-OH, Boc-Trp(CHO)-OH. Boc-*S*-4-methoxybenzyl selenocysteine was obtained from ChemPep Inc (Wellington, FL, USA)

2. General: HPLC and LC-MS.

Peptides were purified using a Dionex (Sunnyvale, California, U.S) Ultimate 3000 system equipped with a Foxy Jr fraction collector using a Gemini C18 column (5 μ; 10.0 x 250 mm) column [Phenomenex (Torrance, California, U.S)] using a flow rate of 5 mL/min and eluted with an appropriate shallow gradient of increasing concentration of acetonitrile containing 0.1% TFA. The solvent system used was A (0.1% TFA in H_2O) and B (0.1% TFA in CH_3CN).

The purity and peptide masses were confirmed by LC-MS [Agilent Technologies (Santa Clara, California, U.S) 1120 Compact LC equipped with a Hewlett Packard (Palo Alto, California, U.S) 1100 MSD mass spectrometer] using ESI in the positive mode. The fractions were monitored using an Agilent Zorbax C3 (3.5 μ; 3.0 x 150 mm) column at 0.3 mL/min with a linear gradient of 5–65%B over 21 min (i.e. 3% B per minute). The solvent system used was A (0.1% formic acid in H_2O) and B (0.1% formic acid in CH_3CN). Fractions were collected, after being analyzed by LC-MS, pooled, and lyophilized.

3. Synthesis of selnocysteine analogue: ^{7}Sec-^{81}Sec-Cp-5 (1).

A. Thioester fragment: ^{1}Gly-^{7}Sec-^{35}Glu-$COSCH_2CH_2$-Ala-OH (2)

Boc-Ala-PAM linker (0.2 mmol) was coupled to aminomethyl resin synthesized as described by Harris *et al.*[1] (loading 1 mmol/g) with DIC (0.4 mmol) in CH_2Cl_2 (3 mL) for 1 h, drained and washed with CH_2Cl_2. The Boc group was removed by treatment with neat TFA and a solution of *S*-trityl-3-mercaptopropionic acid (5 equiv.) /HATU (5 equiv) and $^{i}Pr_2EtN$ (12 equiv) was added and the mixture stood for 20 min. The

trityl group was deprotected using TFA/TIPS/H_2O (95/2.5/2.5, *v/v/v*) for 2 min. and Boc-Glu-(OcHex)-OH (0.4 mmol) was coupled using HBTU (0.39 mol)/ *i*Pr2EtN (1 mmol) in 0.95 mL DMF for 1 h. The remaining amino acids were coupled using the Boc *in situ* neutralisation procedure[2] using 100% TFA as deblocking reagent (1 x 2 min) and HATU/*i*Pr_2NEt as coupling reagent (1 x 5 min). Boc-*S*-4-methoxybenzyl selenocysteine was coupled using HATU/*i*Pr_2EtN for 15 min. Following chain assembly, concomitant reduction of Met(O) and final Boc group removal was carried out on resin using NH_4I/TFA/Me_2S as described by Vilaseca *et al.*[3] The crude peptides **2** was cleaved from the resin with simultaneous removal of side chain protecting groups using HF/*p*-cresol (20:1, *v/v*) for 1 h at 0°C. Following evaporation of HF, the peptides were precipitated with cold diethyl ether, isolated by centrifugation, washed twice with cold diethyl ether, dissolved in 1:1 (*v/v*) CH_3CN/H_2O containing 0.1% TFA, filtered, and lyophilized. Purification using a slow gradient of 0.1 % CH_3CN per min on a Gemini C18 column (5 μ; 10.0 x 250 nm) as described by Harris *et al.*[4] afforded **2** (53 mg, 38% yield).

B. Cysteinyl fragment: ^{36}Cys-^{81}Sec-^{82}Pro-COOH (3)

Boc-Pro-PAM linker (0.2 mmol) was coupled to aminomethyl resin (0.1 g for 0.1 mmol scale, loading 1 mmol/g) with DIC (0.4 mmol) in CH_2Cl_2 (3 mL) for 1 h, drained and washed with CH_2Cl_2. The peptide was assembled using the Boc *in situ* neutralisation procedure[2] using 100% TFA as deblocking reagent (1 x 2 min) and HATU/*i*Pr_2NEt as coupling reagent (1 x 5 min). Boc-*S*-4-methoxybenzyl selenocysteine was coupled using HATU/*i*Pr_2EtN for 15 min. Following chain assembly, the crude peptide **3** was cleaved from the resin as described above to afford crude **3**, which was purified with a slow gradient of 0.1 % CH_3CN per min on a Gemini C18 column (5 μ; 10.0 x 250 nm) as described by Harris *et al.*[4] to give **3** (65 mg, 43% yield).

Both fragments **1** and **2** were aliquot into 1 mg sample to avoid selenol oxidation when handled.

4. Native chemical ligation of selenocysteine analogue Cp-5.

The two reactants, thioester fragment **2** and cysteinyl fragment **3** were dissolved in a solution of 6 M Gd.HCl, 0.2 M Na_2HPO_4 (pre degassed with argon) containing 1 % *v/v* PhSH / at a final peptide concentration of 5 mM, and the pH was readjusted to 7.5. The vial was capped under argon and the reaction mixture was left stirring at room temperature. A 2 μL aliquot of the reaction mixture was diluted four-fold into an aqueous solution (5% TFA *v/v*) for LC-MS analysis. After 10 h the reaction reached completion and was quenched by the addition of 0.2 M Na_2HPO_4 at different pH (5, 3 and 2) to optimize the purification. The crude ligation product was purified by RP-HPLC to yield ligated peptide **4** (32% yield) [$(M+10H)^{+10}$ observed = 921.2 Da; $(M+10H)^{+10}$calculated reduced = 922.0 Da].

5. Protein folding.

The protein was refolded for 4 hours in a buffer containing 50 mM Tris-HCl, pH 7.4, 150 mM NaCl and 2 mM oxidised glutathione at 4 °C (no reduced glutathione was used to avoid any deselenization reaction). Precipitated protein was removed by centrifugation at 6,000 g at 4 °C, for 15 min. This solution was purified by size exclusion chromatography.

6. Size-exclusion chromatography.

Size-exclusion chromatography at 4 °C was used as the final purification step to isolate [^{7}**Sec-**^{81}**Sec]-Cp-5 (1)** as a monomer. The protein was loaded at 1 mL/min onto a Hi-Load Superdex 75 prep grade (10/30) column (GE Healthcare), which was connected to an ÄKTA-FPLC system controlled by the UNICORN software (GE Healthcare). The column was pre-equilibrated with 2 column volumes of 20 mM sodium phosphate at pH 5.2, the optimum pH of the native protein. The elution profile was monitored by measurement of UV-absorbance at 280 nm (Figure *S1*). The folded analogue [^{7}**Sec-**^{81}**Sec]-Cp-5 (1)** was identified by mass spectrometry to be present in fractions 14-16 hence they were combined together (2 mg, 35 %). A shoulder peak was also observed (fractions 17-18) that was established to be by-products resulting from the folding reaction.

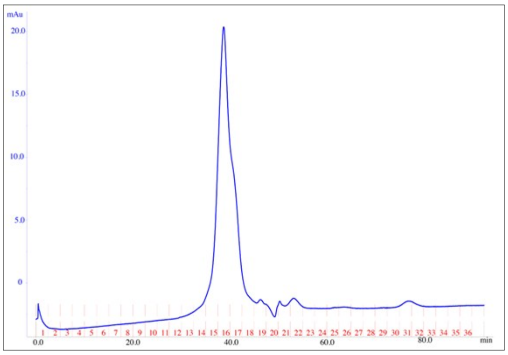

Figure *S1*. Chromatogram of the size-exclusion purification at a 1 mL / min flow rate. The absorbance (milli-absorbance units) of the flow-through was monitored at 280 nm against time (min). Fractions 14-16 contained [**^{7}Sec-^{81}Sec]-Cp-5 (1).**

7. Circular Dichroism.

All CD measurements were performed on a PiStar spectrometer [Applied Photophysics (Leatherhead, UK)]. Protein spectra data are reported in terms of the mean residue ellipticity (θ) (deg.cm^2/dmol), calculated as follows:[5]

$$\theta = S / (10 \times c \times L \times n)$$

S is the raw CD signal in millidegrees, c is the protein concentration (M), L is the cuvette path length (cm), and n is the number of peptide bonds in the protein. The CD spectrum measurement represents the average of seven scans obtained with a 2 nm optical bandwidth. Baseline spectra were collected with buffer alone and then subtracted from the raw protein spectra. The measurements were performed at protein concentrations of 5 µM in 20 mM sodium phosphate buffer, pH 5.2 at 25 °C in 1 mm quartz cuvettes [Hellma Analytics, (Müllheim, Germany)]. Thermal transition experiments were recorded using peptide solutions at 10 µM in 20 mM sodium phosphate buffer, pH 5.2 that had been incubated at 5 °C for a minimum of 24 hours. The solutions were heated from 5 to 80 °C in 2 °C steps with a three minute equilibration time at each step.

8. Fluorescence quenching dependent on ΔpH

Proton translocation into inverted membrane vesicles was measured by the quenching of the fluorescent, pH-dependent probe, acridine orange (AO) using a Cary Eclipse Fluorescence spectrophotometer. The assay buffer contained 10 mM HEPES (pH 6.0), 100 mM KCl, 5 mM $MgCl_2$, 0.085 mg/mL *E. coli* inverted membrane vesicles and 2.5 μM AO. The reactions were initiated with 50 μM NADH and quenching reversed as indicated (R). The excitation and emission wavelengths were 493 and 530 nm, respectively.

9. References.

1. P. W. R. Harris, S. H. Yang and M. A. Brimble, *Tetrahedron Lett*, 2011, **52**, 6024-6026.
2. M. Schnolzer, P. Alewood, A. Jones, D. Alewood and S. B. H. Kent, *Int. J. Pept. Res. Ther.*, 2007, **13**, 31-44.
3. M. Vilaseca, E. Nicolás, F. Capdevila and E. Giralt, *Tetrahedron*, 1998, **54**, 15273-15286.
4. P. W. R. Harris, D. J. Lee and M. A. Brimble, *Journal of Peptide Science*, 2012, **18**, 549-555.
5. S. M. Kelly, T. J. Jess and N. C. Price, *Biochimica et Biophysica Acta (BBA)-Proteins and Proteomics*, 2005, **1751**, 119-139.
